# Incremental Effectiveness of a Second Varicella Vaccine in Children: A Prospective Cohort Study in Anhui, China

**DOI:** 10.3390/vaccines14060544

**Published:** 2026-06-20

**Authors:** Kun Xuan, Tao Li, Zhenqiu Zha, Shujie Zhou, Feiyang Song, Yu Chai, Xianwei Luo, Xingya Pang, Qingru Li, Fanhong Meng, Zuozhi Xiang, Chaoyin Zhu, Tao Wang, Haiyan Wu, Xiaofeng Huang, Yang Li, Jihai Tang

**Affiliations:** 1 Anhui Provincial Center for Disease Control and Prevention, Hefei 230601, China; kunxuan0723@163.com (K.X.);; 2 Sinovac (Dalian) Vaccine Technology Co., Ltd., Dalian 116620, China; 3 Sinovac Holding Group Co., Ltd., Beijing 100085, China; 4 Funan County Center for Disease Control and Prevention, Fuyang 236300, China; 5 Huoqiu County Center for Disease Control and Prevention, Lu’an 237499, China; 6 Lai’an County Center for Disease Control and Prevention, Chuzhou 239200, China; 7 Susong County Center for Disease Control and Prevention, Anqing 246500, China; 8 Ningguo City Center for Disease Control and Prevention, Xuancheng 242000, China

**Keywords:** varicella vaccine, SV-1 cell line, two-dose varicella vaccination schedule, vaccine effectiveness, incremental vaccine effectiveness

## Abstract

**Background:** Varicella remains a common vaccine-preventable disease in China. Although Anhui Province recommended a two-dose varicella vaccine (VarV) schedule in 2021, real-world evidence on the incremental benefit of the second dose is limited. **Methods:** A prospective cohort study among children aged 1–12 years was conducted in Anhui Province from July 2022 to August 2025. Children aged 1–3 years who had received one dose of the human diploid cell line-based (SV-1) VarV and children aged 4–12 years whose second dose was the SV-1 VarV were enrolled in the exposed group and were compared with children who had no history of VarV and those who had received only one dose of the VarV, respectively. Varicella cases were collected through active follow-up and surveillance systems. Vaccine effectiveness (VE) and incremental VE were estimated as [1 − relative risk (RR)] × 100%, where the RRs were calculated based on the incidence densities of breakthrough varicella. **Results:** Overall, 50,054 participants were finally enrolled, contributing 125,351.5 person-years and 105 valid cases. The VE in children aged 1–3 years was 79.1% (95%CI: 42.8–92.4%). Among children aged 4–12 years, the incremental VE was 65.0% (95%CI: 41.9–78.9%), with incremental VEs of 60.1% (95%CI: 22.3–79.5%) for ages 4–6 years and 72.7% (95%CI: 37.8–88.0%) for ages 7–12 years. **Conclusions:** One-dose SV-1 VarV provided substantial protection in young children, and a second dose conferred significant additional protection in children aged 4–12 years, supporting strengthened implementation of the two-dose strategy and catch-up vaccination among school-aged children.

## 1. Introduction

Varicella-zoster virus (VZV) is a highly human-specific alpha herpesvirus; primary infection causes varicella (or chickenpox), which is clinically characterized by a generalized, polymorphic rash [1]. Following infection, the virus establishes latency in the nerve ganglia and may reactivate years later, causing herpes zoster. It has been reported that the lifetime prevalence of herpes zoster is approximately 30% [2]. Varicella is highly contagious, and the population is generally susceptible to it. It is prone to causing clusters or outbreaks in childcare facilities and schools, and is one of the major causes of public health emergencies in educational settings. Although varicella is typically a mild, self-limiting disease, severe cases may lead to complications such as pneumonia and encephalitis and can be fatal. The World Health Organization (WHO) estimates that varicella is associated with approximately 4.2 million hospitalizations due to severe complications and 4200 deaths globally each year, imposing substantial health losses and a public health burden [3].

Vaccination with the live attenuated varicella vaccine (VarV) is the most cost-effective and effective measure to prevent varicella. The WHO recommends VarV for children aged ≥ 1 year [3]. As of 2025, 51 WHO member states or territories have introduced varicella vaccination into routine immunization programs, and varicella incidence and outbreaks have declined markedly compared with the pre-vaccine era [4]. However, VarV has long been managed as a non-National Immunization Program vaccine in China, and there are significant variations in immunization strategies and vaccination coverage across different regions, making varicella one of the most prevalent vaccine-preventable infectious diseases in the country. Evidence indicates that a single dose of VarV provides a high level of protection against moderate-to-severe varicella in preschool- and elementary-aged children [5], but the overall effectiveness is limited and may wane over time, particularly in children ≥ 6 years, which allows breakthrough infections and continued transmission in high-exposure settings [6,7]. In contrast, a two-dose schedule increases protection in children aged 4–11 years and reduces breakthrough infections [8,9,10]. Substantial reductions in varicella incidence among people aged 0–19 years and 0–14 years have been observed after implementing a two-dose strategy in Beijing, Guangzhou, and Shenzhen [11,12]. A systematic review and meta-analysis suggest that two doses provide an additional 63–81% protection for children compared with one dose [13]. In China, the reported incremental vaccine effectiveness (VE) of the second dose among children eligible for a two-dose VarV schedule ranges from 47% to 75% across settings [10,14,15]. Given the heterogeneity in study designs, case ascertainment, and follow-up intensity, further real-world evaluations across different regions remain necessary.

Before 2021, Anhui Province adopted a single-dose VarV vaccination strategy. Since June 2021, a two-dose schedule has been recommended across the province (the first dose is administered after 12 months of age; the second dose is administered after 4 years of age; catch-up vaccination is done as early as possible for those not vaccinated on time, with a minimum interval of 3 months). Following implementation, the local real-world evidence remains limited for quantifying the incremental protection conferred by the second (booster) dose among children who previously received one dose, and the prospective evidence on durability is also lacking. Currently, VarV is predominantly used globally as a monovalent formulation or in combination with the measles, mumps, and rubella vaccine. A VarV manufactured by Sinovac (Dalian) Vaccine Technology Co., Ltd. is prepared using an attenuated VZV (Oka strain) strain and human diploid cells (SV-1). Each dose of SV-1 VarV contains 0.5 mL, with a viral titer > 3.3 lgPFU per dose within its shelf life. The vaccine was approved for marketing in China in 2019 and received WHO prequalification in 2022 [16]. Clinical trials and post-marketing studies demonstrated that SV-1 VarV exhibits favorable efficacy, immunogenicity, and reactogenicity [17,18]. However, with the widespread introduction of SV-1 VarV, no post-licensure effectiveness study has yet been conducted in real-world settings.

Therefore, this study conducted a prospective open-cohort study across multiple regions of Anhui Province from July 2022 to August 2025, aiming to evaluate (1) the VE of a single dose of SV-1 VarV among children aged 1–3 years; and (2) the incremental VE of a second dose of SV-1 VarV among children aged 4–6 years and 7–12 years compared with the single-dose schedule, thereby providing evidence to evaluate and optimize the provincial two-dose VarV strategy.

## 2. Materials and Methods

### 2.1. Study Design and Inclusion Criteria

This prospective open-cohort study was conducted among children aged 1–12 years in five cities of Anhui province: Fuyang, Lu’an, Chuzhou, Anqing, and Xuancheng. This study enrolled participants in an approximately 1:1 ratio between July 2022 and December 2022, dividing them into an exposed group and a control group based on vaccination history. All participants were enrolled in this study after obtaining informed consent. The endpoint of follow-up was the onset of varicella. Person-time was accrued from cohort entry until varicella onset, loss to follow-up, out-migration, or violation of the eligibility criteria due to vaccination history.

The eligibility criteria were as follows: (1) for the exposed group, children aged 1–3 years with a history of one dose of SV-1 VarV, or children aged 4–6 years and 7–12 years with a history of two doses of VarV where the second dose was SV-1 VarV; (2) for the control group, children aged 1–3 years had no history of VarV vaccination, or children aged 4–6 years and 7–12 years with a history of only one dose of VarV; (3) children aged 1–12 years with no prior history of varicella infection and being otherwise healthy; and (4) children who had been long-term residents of the study area.

### 2.2. Follow-Up of the Cohort

The first follow-up was conducted during October to December 2022, during which the baseline information collected included demographic information, vaccination information and varicella history. Thereafter, active follow-up was performed every three months to record varicella onset and any new VarV vaccination history of participants through parental reports. Concurrently, these two sets of information were regularly retrieved and verified from the China Information System for Disease Control and Prevention [19] and the Anhui Provincial Immunization Information Management System. Participants were followed up until varicella onset, loss to follow-up, out-migration, or a change in vaccination history that violated the eligibility criteria.

During follow-up, children who met the inclusion criteria for the exposed group were enrolled as they became eligible. If children in the control group subsequently received VarV and met the criteria for the exposed group (or for an age-specific control group), they were reclassified accordingly and continued to be followed.

### 2.3. Case Definition

Varicella cases were classified as clinically diagnosed cases or laboratory-confirmed cases. Breakthrough cases detected 42 days or more after the most recent VarV dose were considered valid cases. Clinically diagnosed cases were defined as those presenting with typical manifestations such as pruritic maculopapular rash and vesicular lesions [20]. Clinically diagnosed cases were identified via parental report and periodic searches in the CISDCP. Laboratory-confirmed cases were defined as either a 4-fold or greater rise in VZV antibodies from the acute to convalescent phase, or a positive VZV nucleic acid test [21]. Laboratory-confirmed cases were identified by collecting acute-phase blood samples within 0–7 days of the onset of new varicella cases and convalescent-phase blood samples within 21–49 days, followed by antibody detection via the fluorescent antibody membrane antigen (FAMA) test, or by performing polymerase chain reaction (PCR) testing on vesicular fluid samples. This study conducted epidemiological investigations on all cases that consented to the collection of acute-phase or vesicular fluid samples.

### 2.4. Statistical Analysis

Data cleaning and statistical analysis were performed using the SAS 9.4 software (SAS Institute Inc., Cary, NC, USA). Each participant was assigned a unique identifier, and personal details (e.g., name and address) were concealed during analysis. Quantitative data were characterized using the mean and standard deviation (SD) as well as the median and interquartile range (IQR), and compared between groups using *t*-tests. Qualitative data were described using frequencies and percentages, and compared using the chi-square test or Fisher’s exact test. The characteristics of cases in the cohort were described, including age, gender, the interval between the last dose of VarV and the onset of the disease, and the interval between the two doses for cases in the 4–12-year exposed group.

The incidences were calculated for different vaccination groups in each age group, and 95% confidence intervals (CIs) were estimated using the Clopper–Pearson method [22]. VE was assessed by calculating the incidence densities across different vaccination groups across each age group. Incidence densities were calculated as the number of cases divided by the cumulative person-years and multiplied by 1000, and 95% CIs were calculated using the Wald method. Poisson regression models with a log link function and log(person-time/1000) as offset were used to estimate the rate ratios (RRs) and their 95% CIs for VE and incremental VE in children aged 1–3 years and 4–12 years, adjusted for group, gender, and age [23]. The intergroup differences between the two groups was obtained using least-squares means. VE was calculated as (1 − RR) × 100%. In the 1–3 years age group, unvaccinated participants served as the reference group for calculating VE, while in the 4–12 years age group, recipients of the one-dose vaccine served as the reference group for calculating the incremental VE. Because this was an open cohort, participants could be reclassified and VE re-evaluated when their vaccination status changed.

In addition, the protective effect at specific time points during the effective monitoring period was assessed based on the duration of the monitoring period. Sensitivity analyses were conducted by (1) analyzing clinically diagnosed cases and laboratory-confirmed cases separately; (2) comparing results across the initial cohort, per-protocol cohort, and intention-to-treat cohort and (3) an analysis of the incremental VE according to the manufacturer of the first VarV dose among children aged 4–12 years. Two comparisons were made: homologous two-dose SV-1 VarV recipients versus one-dose SV-1 VarV recipients, and heterologous second-dose SV-1 VarV recipients versus one-dose non-SV-1 VarV recipients. A two-sided *α* = 0.05 was used, and *p* < 0.05 was considered statistically significant.

## 3. Results

### 3.1. Cohort Overview

**Figure 1:**
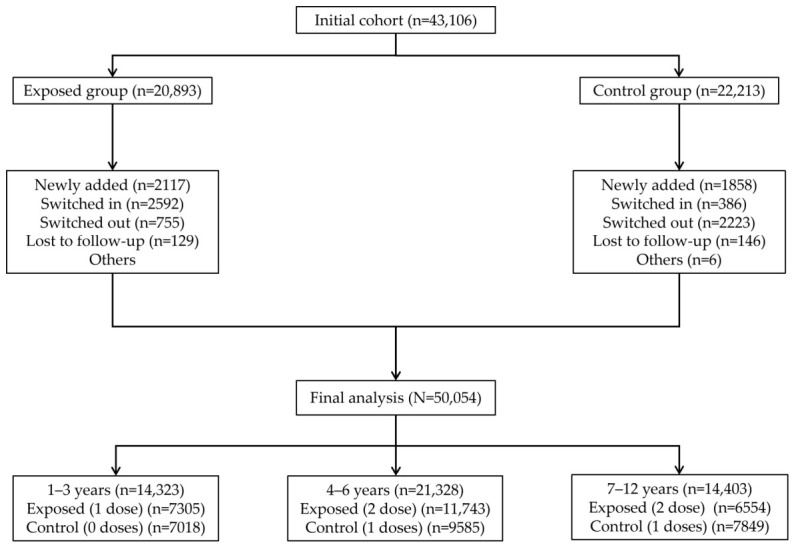
Flow diagram of the study.

A total of 43,106 children were enrolled between July and December 2022, and an additional 3975 participants entered the cohort during follow-up. In addition, 2978 participants changed their exposed category during follow-up. By the end of August 2025, a total of 50,059 person-times were included, and after excluding five cases that occurred outside the valid surveillance period, 50,054 person-times were included in the final analysis, with 25,602 in the exposed group and 24,452 in the control group. There were 14,323 children aged 1–3 years, with 7305 in the exposed group and 7018 in the control group; 21,328 children aged 4–6 years, with 11,743 in the exposed group and 9586 in the control group; and 14,403 children aged 7–12 years, with 6554 in the exposed group and 7853 in the control group. For details on the study protocol, see Figure 1 and Table S1.

In the 1–3-year group, the mean age was 1.8 (0.7) years in the exposed group and 2.2 (0.9) years in controls, with male proportions of 54.5% and 54.4%, respectively. In the 4–6-year group, the mean age was 5.3 (0.9) vs. 5.6 (1.0) years, with male proportions of 52.9% vs. 53.9%, respectively. In the 7–12-year group, the mean age was 9.3 (1.6) vs. 9.9 (1.7) years, with male proportions of 52.6% vs. 54.7%, respectively (Table 1).

### 3.2. Varicella Incidence

A total of 105 valid cases were identified during the study period, and the overall incidence was 2.10 per 1000 (95% CI: 1.72–2.55). The incidence by vaccination status was 2.99 per 1000 (95% CI: 1.85–4.56) in the zero-dose group, 2.55 per 1000 (95% CI: 1.96–3.19) in the one-dose group, and 1.15 per 1000 (95% CI: 0.71–1.75) in the two-dose group; the corresponding incidence density per 1000 person-years was 1.38 (95% CI: 0.90–2.11), 0.96 (95% CI: 0.75–1.22), and 0.44 (95% CI: 0.28–0.67), respectively. Overall, males accounted for a higher proportion of cases (zero doses: 66.7%; one dose: 50.8%; two doses: 57.1%). Among vaccinated cases, the median time since the last dose was 2760.0 (2364.0) days in the one-dose group and 481.0 (602.0) days in the two-dose group; among two-dose recipients, the median interval between the two doses was 1516.0 (1104.5) days (Table 2). Additionally, acute-phase blood samples were collected from 27 cases during the follow-up period, of which 19 were laboratory-confirmed, while the remaining 8 cases were classified as clinically diagnosed due to the absence of convalescent-phase samples. The symptoms in both case types were predominantly mild (68.4% vs. 87.5%), with no significant differences in other clinical manifestations (Table S2).

### 3.3. VE and Incremental VE

The total follow-up time was 125,351.5 person-years in this study, with a mean follow-up duration of 30.0 months per participant. The exposed group contributed 66,644.8 person-years (mean follow-up 31.2 months), and controls contributed 58,706.7 person-years (mean follow-up 28.8 months) (Table 3).

#### 3.3.1. VE of a Single Dose of VarV

In the 1–3-year group, the total follow-up time was 33,683.6 person-years (mean 28.3 months), including 18,442.4 person-years in the exposed group and 15,241.2 person-years among controls. The incidence density was 0.27 per 1000 person-years (95% CI: 0.11–0.65) and 1.38 per 1000 person-years (95% CI: 0.90–2.11), respectively. The adjusted RR was 0.20 (95% CI: 0.07–0.52), corresponding to the VE of 79.1% (95% CI: 42.8–92.4%).

#### 3.3.2. Incremental VE of Two Doses of VarV

In the 4–12-year group, the total follow-up was 91,667.9 person-years (mean 30.7 months), including 48,202.4 person-years in the exposed group and 43,465.5 person-years among controls. The incidence density was 0.44 per 1000 person-years (95% CI: 0.28–0.67) and 1.33 per 1000 person-years (95% CI: 1.03–1.73), respectively. The adjusted RR was 0.35 (95% CI: 0.21–0.58), and the incremental VE was 65.0% (95% CI: 41.9–78.9%).

After stratification by age, the 4–6-year group contributed 52,396.4 person-years (mean 29.5 months). The incidence densities for the two groups were 0.47 per 1000 person-years (95% CI: 0.28–0.79) and 1.08 per 1000 person-years (95% CI: 0.72–1.61). The adjusted RR was 0.40 (95% CI: 0.20–0.78), corresponding to an incremental VE of 60.1% (95% CI: 22.3–79.5%).

The 7–12-year group contributed 39,271.6 person-years (mean 32.8 months). The incidence densities for the two groups were 0.39 per 1000 person-years (95% CI: 0.18–0.81) and 1.61 per 1000 person-years (95% CI: 1.15–2.25). The adjusted RR was 0.27 (95% CI: 0.12–0.62), corresponding to an incremental VE of 72.7% (95% CI: 37.8–88.0%).

### 3.4. Time-Specific Effectiveness

At 12 months after the start of surveillance, the VE of a single dose vaccine became statistically significant (74.96%, 95% CI: 21.17–92.05%) and peaked at 24 months (81.94%, 95% CI: 44.87–94.08%). The additional protection of the two-dose vaccine was also observed at 12 months (59.27%, 95% CI: 14.27–80.64%), peaked at 24 months (70.14%, 95% CI: 42.48–84.50%), and declined slightly by 36 months but remained higher than the level observed at 12 months (64.99%, 95% CI: 41.89–78.91%) (Table 4).

### 3.5. Sensitivity Analyses

Across age groups, SV-1 VarV showed significant protection against clinically diagnosed varicella, with effectiveness estimates of 83.3% (95% CI: 49.8–94.4%) in the 1–3-year group, 61.1% (95% CI: 14.9–82.2%) in the 4–6-year group, and 65.3% (95% CI: 19.2–85.1%) in the 7–12-year group. The incremental VE against laboratory-confirmed cases was significant in the 4–12-year group (73.5%, 95%CI: 18.2–91.4%). Furthermore, analyses in the initial cohort, the per-protocol cohort and the intention-to-treat cohort were consistent with the primary analysis. When stratified by the type of the first VarV dose, the incremental VE was significant only for the group receiving SV-1 VarV as the second dose among children aged 4–12 years (66.5%, 95%CI: 43.6–80.1%) (Table S3).

## 4. Discussion

There is now broad global consensus that a two-dose VarV schedule is more effective than a single dose. To our knowledge, this study provides the first real-world evidence from Anhui Province to quantify incremental VE in children aged 4–12 years and to evaluate the implementation impact of the two-dose strategy, which is important for varicella prevention and control in Anhui. Furthermore, this study represents the first post-licensure evaluation of the protective effectiveness of SV-1 VarV in real-world settings. In this prospective open-cohort study, we confirmed the real-world effectiveness of VarV and highlighted the incremental value of administering the second dose during the school-age years. The results showed that a single dose of SV-1 VarV provided substantial protection (VE: 79.1%, 42.8–92.4%) among children aged 1–3 years, which was comparable to the VE observed in phase III clinical trials among children aged 1–4 years (80.2%, 95% CI: 10.7–95.6%) [17]. However, this level of protection may not fully cover the entire risk period across childhood. As children enter group settings with higher exposure intensity and as the time since vaccination lengthens, breakthrough infections may become more likely among one-dose recipients. Therefore, it is clearly beneficial for children to receive their second dose of the vaccine before or during school entry. In children aged 4–12 years, a second dose of SV-1 VarV provides an additional 65.0% (95% CI: 41.9–78.9%) protection compared with one dose, which is consistent with findings from previous studies on the incremental effectiveness of two-dose VarV in Germany, South Korea, and multiple regions in China [10,14,15,24,25]. Additionally, a previous study indicated that the post-marketing adverse reactions of SV-1 VarV are lower than those of other VarV, confirming its favorable safety profile [18]. Together, these results reinforce the effectiveness of the SV-1 VarV under real-world conditions and support its broader implementation.

Notably, the incremental VE in the 4–6-year group (60.1%, 95% CI: 22.3–79.5%) was lower than that in the 7–12-year group (72.7%, 95% CI: 37.8–88.0%). This age difference may be explained by variations in the time since primary vaccination and exposure intensity. In older children with a longer time since the first dose, antibody levels may have fallen below the protective thresholds, leading to a mismatch between immune protection and exposure intensity, thereby increasing the risk of breakthrough infection [26]. In contrast, younger children may experience less immune waning and lower exposure intensity; consequently, although some individuals may exhibit primary vaccine failure or a suboptimal response to the first dose [27,28], the overall incremental benefits of the second dose may still be limited at the population level. The second dose could strengthen immune memory, enabling the body to activate a specific immune response more rapidly and effectively upon re-exposure, thereby reducing disease risk [29]. The incremental effect of the second dose of VarV is consequently amplified through adaptive protection, which balances immune strength against exposure intensity in the 7–12-year group. By using one-dose recipients as the controls, this study directly quantified the incremental contribution of the second dose, aligning with a practical policy question: the expected epidemiological benefits from administering a booster among children who have already received one dose of VarV.

The incremental VE of the second dose peaked at 24 months after surveillance initiation. This pattern likely reflects the combined influence of exposure intensity and cohort dynamics. During early follow-up, limited case accrual makes estimates more vulnerable to random variation; as follow-up extends into the second year, exposure opportunities increase and case numbers stabilize, improving the ability to detect differences in breakthrough risk between two-dose and one-dose recipients and resulting in an apparent peak around 24 months [30]. Children transition into daycare or school and experience term-to-term mixing, expanding contact networks and increasing exposure pressure [31]; under these conditions, one-dose recipients may be more likely to experience a mismatch between immune protection and exposure intensity, thereby magnifying the relative advantage of two doses. Overall, although the incremental VE at 36 months was slightly lower than at 24 months, it remained high, supporting that SV-1 VarV as a booster dose sustains significant protective benefits over at least a three-year observation period.

Sensitivity analyses supported the robustness of our findings. The results were consistent across different analytic cohorts, with no material changes in effect direction or statistical significance. Although the analysis of laboratory-confirmed cases did not show clear efficacy for two doses of VarV, which may be limited by the fact that not all cases consented to sample collection and the sensitivity of case identification, two-dose VarV showed significant protection against clinically diagnosed cases across age groups. Previous studies have shown that breakthrough cases following vaccination typically present with milder symptoms [32,33]. Given that the majority of participants in this study had received at least one or even two doses of the VarV, the potential for clinical under-ascertainment or misclassification of mild cases cannot be excluded, which may have led to an inaccurate assessment of VE. However, no significant differences in clinical manifestations were observed between the two types of cases in this study. Although the small sample size of clinically diagnosed cases limits their representativeness, these results nonetheless partially ruled out the potential impact of cases with different severity on VE estimates. Additionally, we performed an exploratory analysis of the incremental VE for homologous and heterologous vaccination. Owing to the late large-scale introduction of SV-1 VarV, older children in this study had not received SV-1 VarV as their first dose. Limited by the number of participants receiving homologous vaccination, the two doses homologous regimen with SV-1 VarV did not show a significant incremental VE; however, this does not imply that homologous vaccination is ineffective, and its efficacy should be evaluated over a longer time scale.

Several limitations should be considered. First, as a real-world prospective cohort, vaccination was not randomly assigned. Although models adjusted for age and gender, residual confounding may persist (e.g., childcare/school attendance status, household socioeconomic status, prior exposure opportunities, and healthcare-seeking and reporting behaviors), potentially biasing incremental VE estimates. Secondly, the case reporting may be incomplete. Varicella is not mandatorily reportable in Anhui, and the incidence observed in this cohort (2.10 per 1000) was substantially higher than the average reported incidence during 2012–2021 (44.8 per 100,000) [34]; although active surveillance was strengthened to reduce under-ascertainment and information bias, mild cases not seeking care or not reported may not be fully captured. Thirdly, due to lack of accurate vaccination coverage data, the indirect effects of coverage on varicella transmission were not assessed, which may introduce uncertainty in effect estimation. The WHO notes that sustained coverage above 80% can effectively control transmission, whereas coverage between 30% and 70% may increase incidence and mortality [3]. High coverage can establish herd immunity, thereby protecting the unvaccinated population [35]; this may confuse two effects: “vaccines inducing antibodies in the body” and “high vaccination coverage leaving the virus with nowhere to spread”. Conversely, if second-dose coverage is low, the observed decline in incidence may be primarily attributable to the partial immunity conferred by the first dose or to random fluctuations, and the success in controlling the disease might be mistakenly attributed to a two-dose vaccination strategy that has not yet been implemented on a large scale. Since VarV vaccination is voluntary and self-paid in Anhui Province, and the two-dose immunization strategy was implemented relatively late, the vaccination rates remain relatively low. Therefore, although the potential impact of vaccination rates on VE cannot be entirely ruled out, the direct comparison of two-dose recipients with single-dose recipients in the same setting still provides evidence of significant public health importance. Future studies should integrate data on the vaccination coverage, health status and socioeconomic status of children, and apply methods such as fine matching or propensity score approaches to further evaluate robustness.

## 5. Conclusions

Following implementation of a two-dose VarV strategy in Anhui Province, we have for the first time quantified the incremental VE of the second dose and validated the effectiveness of SV-1 VarV in real-world applications. Overall, the first dose provided substantial direct protection in young children; however, a single-dose strategy may not sufficiently cover the full childhood risk window. Promoting catch-up or routine administration of the second dose of VarV among children aged 4–12 years is therefore expected to yield clear epidemiological benefits. Future work should account for indirect effects related to vaccination coverage, and should also evaluate cost-effectiveness to support policy decisions regarding immunization strategy optimization and resource allocation.

## Figures and Tables

**Table 1:** Demographic characteristics of the cohort.

Characteristic	Exposed Group (N = 25,602)	Control Group (N = 24,452)	Total	*t*/ *χ*^2^	*p*
1–3 years, n	7305	7018	14,323		
Age (years), mean (SD)	1.8 (0.7)	2.2 (0.9)	2.0 (0.8)	−27.54	<0.001
Age (years), median (IQR)	1.6 (1.3, 2.1)	2.0 (1.3, 2.9)	1.7 (1.3, 2.6)		
Male, n (%)	3981 (54.5)	3819 (54.4)	7800 (54.5)	0.01	0.924
Female, n (%)	3324 (45.5)	3199 (45.6)	6523 (45.5)		
4–6 years, n	11,743	9585	21,328		
Age (years), mean (SD)	5.3 (0.9)	5.6 (1.0)	5.5 (0.9)	−25.38	<0.001
Age (years), median (IQR)	5.2 (4.5, 6.1)	5.8 (4.8, 6.5)	5.5 (4.6, 6.3)		
Male, n (%)	6218 (52.9)	5170 (53.9)	11,388 (53.4)	2.07	0.150
Female, n (%)	5525 (47.1)	4415 (46.1)	9940 (46.6)		
7–12 years, n	6554	7849	14,403		
Age (years), mean (SD)	9.3 (1.6)	9.9 (1.7)	9.6 (1.7)	−23.72	<0.001
Age (years), median (IQR)	9.0 (7.9, 10.5)	10.1 (8.5, 11.3)	9.6 (8.1, 11.0)		
Male, n (%)	3447 (52.6)	4291 (54.7)	7738 (53.7)	6.19	0.013
Female, n (%)	3107 (47.4)	3558 (45.3)	6665 (46.3)		
4–12 years, n	18,297	17,434	35,731		
Age (years), mean (SD)	6.7 (2.2)	7.6 (2.5)	7.1 (2.4)	−33.10	<0.001
Age (years), median (IQR)	6.2 (4.9, 8.1)	6.9 (5.6, 9.8)	6.6 (5.1, 8.9)		
Male, n (%)	9665 (52.8)	9461 (54.3)	19,126 (53.5)	7.49	0.006
Female, n (%)	8632 (47.2)	7973 (45.7)	16,605 (46.5)		

SD, standard deviation; IQR, interquartile range.

**Table 2:** Characteristics of varicella cases.

Characteristic	0 Doses (N = 21)	1 Dose (N = 63)	2 Doses (N = 21)
Incidence, per 1000 population	2.99 (1.85, 4.56)	2.55 (1.96, 3.25)	1.15 (0.71, 1.75)
Incidence density, per 1000 person-years	1.38 (0.90, 2.11)	0.96 (0.75, 1.22)	0.44 (0.28, 0.67)
Year, n (%)			
2022	3 (14.3)	8 (12.7)	2 (9.5)
2023	10 (47.6)	26 (41.3)	7 (33.3)
2024	7 (33.3)	18 (28.6)	6 (28.6)
2025	1 (4.8)	11 (17.4)	6 (28.6)
Age (years), mean ± SD	3.20 ± 1.29	9.07 ± 3.27	8.08 ± 2.65
Sex, n (%)			
Male	14 (66.7)	32 (50.8)	12 (57.1)
Female	7 (33.3)	31 (49.2)	9 (42.9)
Time since last dose (days), median (IQR)	-	2760.0 (2364.0)	481.0 (602.0)
Interval between two doses (days), median (IQR)	-	-	1516.0 (1104.5)

SD, standard deviation; IQR, interquartile range.

**Table 3:** Effectiveness of the varicella vaccine.

Group	N	Cases	Incidence ^a^	Person-Years	Incidence Density (95% CI) ^b^	RR (95% CI) ^c^	VE, % (95% CI)	*p*
Age 1–3 years								
Control	7018	21	2.99	15,241.2	1.38 (0.90–2.11)	Ref.	–	–
Exposed	7305	5	0.68	18,442.4	0.27 (0.11–0.65)	0.20 (0.07–0.52)	79.1 (42.8–92.4)	0.002
Age 4–6 years								
Control	9585	24	2.50	22,291.7	1.08 (0.72–1.61)	Ref.	–	–
Exposed	11,743	14	1.19	30,104.7	0.47 (0.28–0.79)	0.40 (0.20–0.78)	60.1 (22.3–79.5)	0.007
Age 7–12 years								
Control	7849	34	4.33	21,173.8	1.61 (1.15–2.25)	Ref.	–	–
Exposed	6554	7	1.07	18,097.8	0.39 (0.18–0.81)	0.27 (0.12–0.62)	72.7 (37.8–88.0)	0.002
Age 4–12 years								
Control	17,434	58	3.33	43,465.5	1.33 (1.03–1.73)	Ref.	–	–
Exposed	18,297	21	1.15	48,202.4	0.44 (0.28–0.67)	0.35 (0.21–0.58)	65.0 (41.9–78.9)	<0.001

a: Incidence is expressed per 1000 population; b: incidence density is expressed per 1000 person-years; c: age and gender were adjusted in the model; RR, rate ratio; CI, confidence interval; Ref., reference; VE: vaccine effectiveness.

**Table 4:** Time-specific vaccine effectiveness following the start of the follow-up in the cohort.

Age Group	Exposed Group			Control Group			VE, % (95% CI) ^b^	*p*
	Cases	Person-Years	Incidence Density (95% CI) ^a^	Cases	Person-Years	Incidence Density (95% CI)		
1–3 years								
D90	0	1798.9	-	3	1670.4	1.80 (0.58–5.57)	-	-
D180	1	3591.5	0.28 (0.04–1.98)	7	3209.3	2.18 (1.04–4.58)	88.93 (8.85–98.66)	0.041
D360	4	7131.7	0.56 (0.21–1.49)	13	5989.3	2.17 (1.26–3.74)	74.96 (21.17–92.05)	0.018
D720	4	13,891.2	0.29 (0.11–0.77)	17	11,092.6	1.53 (0.95–2.47)	81.94 (44.87–94.08)	0.003
D1080	5	18,442.4	0.27 (0.11–0.65)	21	15,241.2	1.38 (0.90–2.11)	79.15 (42.76–92.40)	0.002
4–6 years								
D90	2	2834.4	0.71 (0.18–2.82)	2	2313.6	0.86 (0.22–3.46)	23.45 (−456.10–89.46)	0.792
D180	2	5616.5	0.36 (0.09–1.42)	4	4523.7	0.88 (0.33–2.36)	63.15 (−104.06–93.34)	0.253
D360	6	11,072.4	0.54 (0.24–1.21)	9	8633.0	1.04 (0.54–2.00)	49.67 (−43.13–82.30)	0.198
D720	8	21,637.3	0.37 (0.18–0.74)	16	16,153.7	0.99 (0.61–1.62)	63.20 (13.02–84.43)	0.023
D1080	14	30,097.5	0.47 (0.28–0.79)	24	22,291.6	1.08 (0.72–1.61)	60.13 (22.32–79.53)	0.007
7–12 years								
D90	1	1613.0	0.62 (0.09–4.40)	8	1915.5	4.18 (2.09–8.35)	78.87 (−70.59–97.38)	0.145
D180	3	3224.1	0.93 (0.30–2.89)	9	3811.6	2.36 (1.23–4.54)	52.75 (−77.75–87.44)	0.267
D360	4	6435.6	0.62 (0.23–1.66)	16	7530.2	2.12 (1.30–3.47)	69.08 (6.25–89.80)	0.038
D720	4	12,809.3	0.31 (0.12–0.83)	23	14,849.9	1.55 (1.03–2.33)	78.02 (35.79–92.48)	0.006
D1080	7	18,097.6	0.39 (0.18–0.81)	34	21,173.8	1.61 (1.15–2.25)	72.68 (37.84–87.99)	0.002
4–12 years								
D90	3	4447.5	0.67 (0.22–2.09)	10	4229.1	2.36 (1.27–4.39)	61.68 (−41.68–92.15)	0.151
D180	5	8840.6	0.57 (0.24–1.36)	13	8335.3	1.56 (0.91–2.69)	55.73 (−26.11–84.46)	0.127
D360	10	17,508.0	0.57 (0.31–1.06)	25	16,163.3	1.55 (1.05–2.29)	59.27 (14.27–80.64)	0.018
D720	12	34,446.6	0.35 (0.20–0.61)	39	31,003.6	1.26 (0.92–1.72)	70.14 (42.48–84.50)	<0.001
D1080	21	48,195.1	0.44 (0.28–0.67)	58	43,465.4	1.33 (1.03–1.73)	64.99 (41.89–78.91)	<0.001

a: Incidence density is expressed per 1000 person-years; b: age and gender were adjusted in the model; CI, confidence interval; VE: vaccine effectiveness.

## Data Availability

The data underlying this article will be available from the corresponding author upon reasonable request.

## Supplementary Materials

The following supporting information can be downloaded at https://www.mdpi.com/article/10.3390/vaccines14060544/s1: Table S1: Enrollment, dynamic reassignment, and case monitoring of the study population. Table S2: Comparison of symptoms between laboratory-confirmed and clinically diagnosed cases of varicella. Table S3: Sensitivity analyses of vaccine effectiveness in the cohort.

## Abbreviations

The following abbreviations are used in this manuscript:

VarV	Varicella vaccine
VE	Vaccine effectiveness
VZV	Varicella-zoster virus
WHO	World Health Organization
SD	Standard deviation
CI	Confidence interval
IQR	Interquartile range
