# Supplementary material for: Incremental Effectiveness of a Second Varicella Vaccine in Children: A Prospective Cohort Study in Anhui, China
Source: Vaccines (Basel). 2026 Jun 20;14(6):544. doi: 10.3390/vaccines14060544 (PMC13308287; doi:10.3390/vaccines14060544)
Supplement: Supplementary file 1 [file vaccines-14-00544-s001.zip › vaccines-4367417-supplementary.pdf]

SUPPLEMENTARY MATERIAL

Supplementary Statistical analysis

Rate ratios (RRs) and their 95% CIs were estimated using Poisson regression models via SAS PROC GENMOD. The comparisons between exposed and control groups were was obtained from least-squares means using LSMEANS/DIFF in PROC GENMOD. When either groups had zero events within a stratum and age group, RR and VE were not estimated to avoid unstable model-based inference.

R version 4.6.0 was used as an independent validation environment, employing the same Poisson regression, offset, and least-squares means contrast logic. The “readxl (v1.5.0)” package was used to read Excel analysis files and SAS output workbooks for validation purposes. The “dplyr (v1.2.1)” package was used for independent data preparation and summarization. The “writexl (v1.5.4)” package was used to generate independent R validation workbooks. The “emmeans (v2.0.3)” package was used to implement Poisson regression models and group comparisons, corresponding to the LSMEANS/DIFF approach in the SAS GENMOD procedure. The results obtained using R were consistent with those generated by the SAS outputs, confirming the reproducibility and robustness of the analytical approach.

Table S1. Enrollment, dynamic reassignment, and case monitoring of the study population

Category	1-3 years			4-6 years			7-12 years			1-12 years		
	Exposed	Control	Total	Exposed	Control	Total	Exposed	Control	Total	Exposed	Control	Total
Total cohort population	7,305	7,018	14,323	11,743	9,586	21,329	6,554	7,853	14,407	25,602	24,457	50,059
Initial cohort[a]	5,745	6,465	12,210	9,434	8,355	17,789	5,714	7,393	13,107	20,893	22,213	43,106
Newly addition[b]	708	553	1261	842	845	1,687	567	460	1,027	2,117	1,858	3,975
Entered from other groups	852	0	852	1,467	386	1,853	273	0	273	2,592	386	2,978
Dynamic group switching												
Switched in	852	0	852	1,467	386	1,853	273	0	273	2,592	386	2978
From the age 1–3 exposure group	0	0	0	755	0	755	0	0	0	755	0	755
From the age 1–3 control group	852	0	852	2	386	388	0	0	0	854	386	1,240
From the age 4–6 control group	0	0	0	710	0	710	176	0	176	886	0	886
From the age 7–12 control group	0	0	0	0	0	0	97	0	97	97	0	97
Switched out	755	1,240	1,995	0	886	886	0	97	97	755	2,223	2,978
To the age 1–3 exposure group	0	852	852	0	0	0	0	0	0	0	852	852
To the age 4–6 exposure group	755	2	757	0	710	710	0	0	0	755	712	1,467
To the age 4–6 control group	0	386	386	0	0	0	0	0	0	0	386	386
To the age 7–12 exposure group	0	0	0	0	176	176	0	97	97	0	273	273
Varicella cases	5	21	26	14	24	38	7	34	41	26	79	105
Laboratory-confirmed cases	1	0	1	4	6	10	0	8	8	5	14	19
Clinically diagnosed cases	4	21	25	10	18	28	7	26	33	21	65	86
Early withdrawal	46	48	94	27	56	83	56	48	104	129	152	281
Migration from the study area	41	19	60	21	22	43	19	7	26	81	48	129
Lost to follow-up	5	27	32	6	34	40	37	41	78	48	102	150
Death	0	2	2	0	0	0	0	0	0	0	2	2

a: Participants enrolled during October–December 2022, excluding those who entered from other groups.

b: Participants enrolled outside October–December 2022, excluding those who entered from other groups.

Table S2. Comparison of symptoms between laboratory-confirmed and clinically diagnosed cases of varicella

Symptoms (%)	Laboratory-confirmed cases (N=19)	Clinically diagnosed cases (N=8)	P^*
Mild (No. of rashes <50)	13 (68.4)	7 (87.5)	0.633
Moderate (No. of rashes 50-100)	6 (31.6)	1 (12.5)	
Fever	4 (21.1)	2 (25.0)	1.000
Headache	2 (10.5)	1 (12.5)	1.000
Sore throat	3 (15.8)	2 (25.0)	0.616
Nausea	1 (5.3)	1 (12.5)	0.513
Stomachache	1 (5.3)	1 (12.5)	0.513

*: The *p*-value is calculated using Fisher's exact test.

Table S3. Sensitivity analyses of vaccine effectiveness in the cohort

Age group	Variable	Exposed group			Control group			RR (95%CI)[b]	VE, % (95%CI)	*P*
		N	Person-years	Incidence density (95%CI)[a]	N	Person-years	Incidence density (95%CI)			
1-3 years	Case definition									
	Laboratory-confirmed cases	1	18,442.4	0.05 (0.01-0.38)	0	15,241.2	-	-	-	-
	Clinically diagnosed cases	4	18,442.4	0.22 (0.08-0.58)	21	15,241.2	1.38 (0.90-2.11)	0.17 (0.06-0.50)	83.3 (49.8-94.4)	0.001
	Analytic cohort									
	Cohort 1[c]	4	13,703.7	0.29 (0.11-0.78)	21	12,932.3	1.62 (1.06-2.49)	0.18 (0.06-0.56)	81.6 (44.3-93.9)	0.003
	Cohort 2[d]	4	15,351.8	0.26 (0.10-0.69)	21	14,224.0	1.48 (0.96-2.26)	0.19 (0.06-0.57)	81.3 (43.4-93.8)	0.003
	Cohort 3[e]	5	17,145.4	0.29 (0.12-0.70)	22	17,660.7	1.25 (0.82-1.89)	0.25 (0.09-0.68)	75.1 (32.3-90.8)	0.006
4-6 years	Case definition									
	Laboratory-confirmed cases	4	30,104.7	0.13 (0.05-0.35)	6	22,291.7	0.27 (0.12-0.60)	0.43 (0.12-1.54)	57.1 (-53.5-88.0)	0.193
	Clinically diagnosed cases	10	30,104.7	0.33 (0.18-0.62)	18	22,291.7	0.81 (0.51-1.28)	0.39 (0.18-0.85)	61.1 (14.9-82.2)	0.018
	Analytic cohort									
	Cohort 1	12	26,516.8	0.45 (0.26-0.80)	22	19,390.4	1.13 (0.75-1.72)	0.37 (0.18-0.76)	62.8 (23.8-81.9)	0.007
	Cohort 2	13	28,290.5	0.46 (0.27-0.79)	24	21,251.8	1.13 (0.76-1.68)	0.37 (0.19-0.74)	62.8 (26.2-81.3)	0.005
	Cohort 3	13	28,290.5	0.46 (0.27-0.79)	24	23,701.2	1.01 (0.68-1.51)	0.42 (0.21-0.84)	57.5 (15.9-78.5)	0.014
	Type of first dose[f]									
	SV-1 VarV	1	797.3	1.25 (0.18-8.90)	4	2100.1	1.90 (0.71-5.08)	0.70 (0.07-6.45)	30.5 (-544.9-92.5)	0.749
	Others VarV	13	29307.3	0.44 (0.26-0.76)	20	20191.7	0.99 (0.64-1.54)	0.41 (0.20-0.83)	59.3 (17.3-79.9)	0.013
7-12 years	Case definition									
	Laboratory-confirmed cases	0	18,097.8	-	8	21,173.8	0.38 (0.19-0.76)	-	-	-
	Clinically diagnosed cases	7	18,097.8	0.39 (0.18-0.81)	26	21,173.8	1.23 (0.84-1.80)	0.35 (0.15-0.81)	65.3 (19.2-85.1)	0.014
	Analytic cohort									
	Cohort 1	7	16,054.1	0.44 (0.21-0.91)	30	19,892.5	1.51 (1.05-2.16)	0.31 (0.14-0.72)	68.7 (28.0-86.3)	0.006
	Cohort 2	7	17,573.3	0.40 (0.19-0.84)	34	21,124.9	1.61 (1.15-2.25)	0.28 (0.12-0.64)	72.0 (36.3-87.7)	0.002
	Cohort 3	7	17,573.3	0.40 (0.19-0.84)	34	21,399.5	1.59 (1.14-2.22)	0.28 (0.12-0.64)	71.6 (35.5-87.5)	0.003
	Type of first dose									
	SV-1 VarV	0	170.8	0.00 (0.00-0.00)	0	2474.5	0.00 (0.00-0.00)	-	-	-
	Others VarV	7	17927	0.39 (0.19-0.82)	34	18699.3	1.82 (1.30-2.54)	0.24 (0.11-0.55)	75.6 (44.5-89.3)	<0.001
4-12 years	Case definition									

Laboratory-confirmed cases	4	48,202.4	0.08 (0.03-0.22)	14	43,465.5	0.32 (0.19-0.54)	0.27 (0.09-0.82)	73.5 (18.2-91.4)	0.021
Clinically diagnosed cases	17	48,202.4	0.35 (0.22-0.57)	44	43,465.5	1.01 (0.75-1.36)	0.38 (0.21-0.67)	62.2 (33.2-78.6)	<0.001
Analytic cohort									
Cohort 1	19	42,570.9	0.45 (0.28-0.70)	52	39,282.9	1.32 (1.01-1.74)	0.36 (0.21-0.61)	64.2 (38.9-79.0)	<0.001
Cohort 2	20	45,863.9	0.44 (0.28-0.68)	58	42,376.7	1.37 (1.06-1.77)	0.34 (0.20-0.57)	65.8 (42.8-79.6)	<0.001
Cohort 3	20	45,863.9	0.44 (0.28-0.68)	58	45,100.7	1.29 (0.99-1.66)	0.36 (0.22-0.61)	63.5 (39.0-78.2)	<0.001
Type of first dose									
SV-1 VarV	1	968.10	1.03 (0.15-7.33)	4	4,574.50	0.87 (0.33-2.33)	0.61 (0.07-5.76)	38.7 (-475.7-93.5)	0.668
Others VarV	20	47,234.30	0.42 (0.27-0.66)	54	38,891.00	1.39 (1.06-1.81)	0.34 (0.20-0.56)	66.5 (43.6-80.1)	<0.001

a: Incidence density is expressed per 1,000 person-years; b: Age and gender were adjusted in the model; c: Cohort 1: Initial cohort; d: Cohort 2: Per-protocol cohort excluding participants who switched groups during follow-up; e: Cohort 3: Intention-to-treat cohort ignoring dynamic group switching; f: Based on the manufacturer of the first VarV dose, two groups were defined: the both-doses SV-1 VarV group and the SV-1 VarV-only-as-second-dose group; RR, rate ratio; CI, confidence interval; Ref., reference; VE: vaccine effectiveness.
